# Computational Cancer Biology: An Evolutionary Perspective

**DOI:** 10.1371/journal.pcbi.1004717

**Published:** 2016-02-04

**Authors:** Niko Beerenwinkel, Chris D. Greenman, Jens Lagergren

**Affiliations:** 1 Department of Biosystems Science and Engineering, ETH Zurich, Basel, Switzerland; 2 SIB Swiss Institute of Bioinformatics, Basel, Switzerland; 3 School of Computing Sciences, University of East Anglia, Norwich, United Kingdom; 4 Science for Life Laboratory, School of Computer Science and Communication, Swedish E-Science Research Center, KTH Royal Institute of Technology, Solna, Sweden; National Cancer Institute, United States of America and Tel Aviv University, Israel, UNITED STATES

Cancer is a leading cause of death worldwide and represents one of the biggest biomedical research challenges of our time. Tumor progression is caused by somatic evolution of cell populations. Cancer cells expand because of the accumulation of selectively advantageous mutations, and expanding clones give rise to new cell subpopulations with increasingly higher somatic fitness (Fig 1). In the 1970s, Nowell and others established this somatic evolutionary view of cancer [1]. Today, computational biologists have the opportunity to take advantage of large-scale molecular profiling data in order to carve out the principles of tumor evolution and to elucidate how it manifests across cancer types. Analogous to other evolutionary studies, mathematical modeling will be key to the success of understanding the somatic evolution of cancer [2].

**Fig 1:**
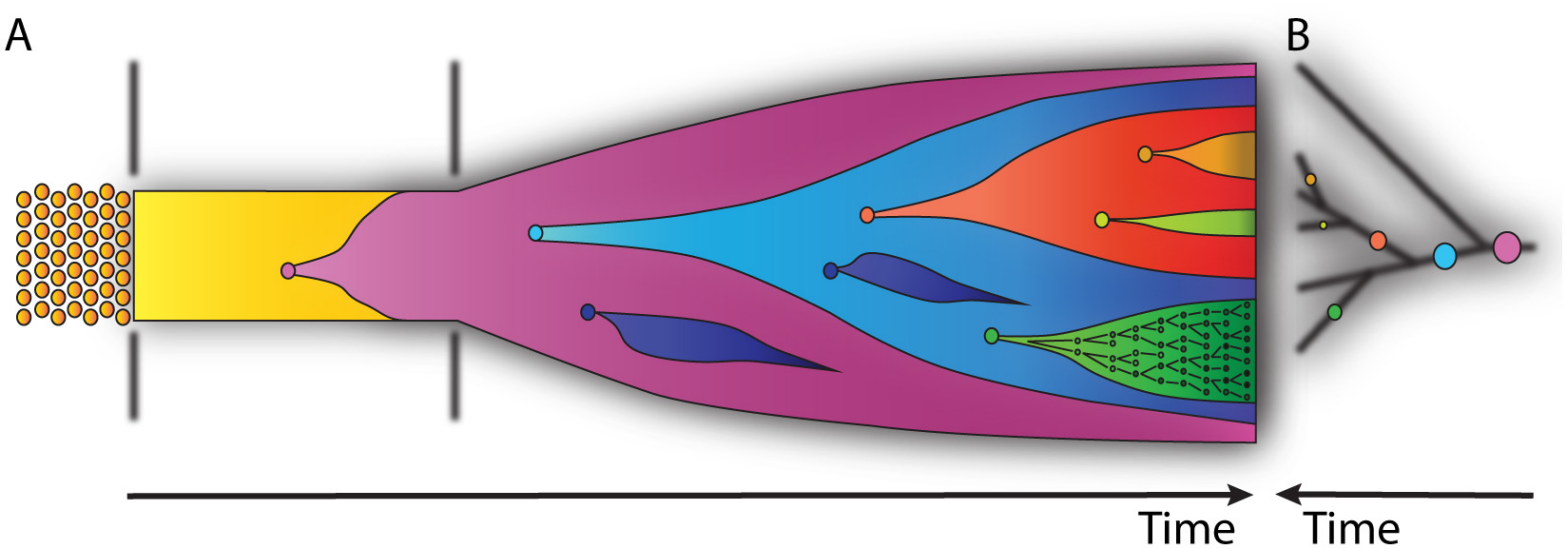
Schematic representation of neoplastic transformation. (A) The left-hand side represents regular homeostatic tissue. The middle region represents a mutation undergoing a selective sweep across a population of phenotypically normal tissue. The right-hand side indicates a period of clonal growth, during which different mutations combine across subclones. (B) A phylogenetic tree on the right mirrors the subclonal structure in (A); the circles represent mutations, and their sizes indicate the size of the corresponding subpopulation. The green subclone contains a branching process of mutation accumulation, indicating the continual stochastic processes that underlie the approximation that is a clonal evolution tree.

In general, cancer research involves a range of clinical, epidemiological, and molecular approaches, as well as mathematical and computational modeling. An early and very successful example of mathematical modeling was the work of Nordling [3] and of Armitage and Doll [4]. In the 1950s, long before cancer genome data was available, they analyzed cancer incidence data and postulated, based on the observed age-incidence curves, that cancer is a multistep process. In search of these rate-limiting events, cancer progression was then linked to the accumulation of genomic alterations. Since then, the evolutionary perspective on cancer has proven useful in many instances, and the mathematical theory of cancer evolution has been developed much further. However, little clinical benefit could be gained from this approach so far. Much of evolutionary modeling in general, and of cancer in particular, has remained conceptual or qualitative, either because of strong simplifications in the interest of mathematical tractability or lack of informative data.

Next-generation sequencing (NGS) technologies and their various applications have changed this situation fundamentally [5]. Today, cancer cells can be analyzed in great detail at the molecular level, and tumor cell populations can be sampled extensively. Driven by this technological revolution, large numbers of high-dimensional molecular profiles of tumors, and even of individual cancer cells, are collected by cancer genome consortia, as well as by many individual labs. Large catalogs of cancer genomes, epigenomes, transcriptomes, proteomes, and other molecular profiles are generated to assess variation among tumors from different patients (intertumor heterogeneity) as well as among individual cells of single tumors (intratumor heterogeneity). These data hold the promise not only of new cancer biology discoveries but also of progress in cancer diagnostics and treatment.

Analyzing these complex data and interpreting them in the context of ongoing somatic evolution, disease progression, and treatment response is a major challenge, and the prospects to improve cancer treatment depend critically on progress with these computational and statistical tasks. In the following, we briefly summarize the current state of the art in the field and highlight major challenges that lie ahead, including (i) reconstruction of evolutionary history based on different types of genomic alterations, (ii) functional interpretation of mutations, and (iii) predictive modeling of the evolutionary dynamics of cancer. We argue that an interdisciplinary approach, including statistical and computational data analysis as well as evolutionary modeling of cancer, will be essential for translating technological advances into clinical benefits.

## Cancer As an Evolutionary Process

Cancer is a genetic disease that arises when normal cellular functions are disrupted by mutations arising in DNA. These changes occur at the level of single cells, which are then propagated into subpopulations as cells divide and pass mutations through cell lineages (Fig 1). Differences in growth rates between clones produce a complex tumor microenvironment consisting of many different interacting and evolving cells, including normal stromal and immune cells. These differences can manifest on various spatial, organizational, and functional levels. Furthermore, although mutations are thought to primarily arise during the development of cancerous tissue, there is a growing body of evidence, including theoretical [6], histological [7], and genetic [8,9] approaches, supporting the idea that somatic mutations occur throughout the entire lifetime of the host organism. Such mutations can be detected at low levels in circulating cells [8], as well as directly from tissue. In eyelid epidermal cells, for example, it has recently been shown that perfectly functional cells harbor a plethora of mutations that are also found in known cancer genes [9].

The resultant intratumor genetic diversity is a huge problem for correctly diagnosing and successfully treating tumors [10,11]. For example, the biopsy obtained from a heterogeneous tumor may not be representative of the entire tumor because of insufficient resolution or spatial heterogeneity. The treatment decision is then based on an incomplete or biased sample and therefore is at risk of failing to target existing but undetected tumor subclones. This problem is particularly pronounced for targeted drug therapy, in which small tumor subpopulations resistant to targeted treatment are likely to preexist in the tumor prior to therapy [12].

Epistatic interactions among mutations are abundant. For example, many different cancer mutations can result in deregulation of the same signaling pathways, and distinct mutational patterns may result in the same phenotype [13,14]. Conversely, the accumulated mutations create an environment that may cause selection to act in temporally and spatially distinct ways [15–19]. For example, a mutation may initially yield a growth advantage, but after growing into a large tumor, the advantage may disappear because the inner regions of the tumor can suffer from necrosis and further growth is impossible without angiogenesis. Whether mutations are adaptive or not will then depend on cellular location within the tumor.

## Molecular Profiling of Tumors

In order to assess tumor diversity and to better understand the evolution of cancer, paired-end sequencing experiments can elucidate the genetic makeup of tumors. A single sample will provide a snapshot of the end result of these evolutionary processes across the cells that are sequenced at that point in time. We would like to use this information to infer the evolutionary history of the tumor, evaluate the rates of mutation and selection, and predict future responses of the tumor to environments potentially controlled by various drug protocols.

The detected mutations can take the form of single-nucleotide variants (SNVs), in which a single nucleotide substitution occurs (or, occasionally, a few consecutive base changes), or structural variants (SVs), in which chunks of DNA are erroneously copied, deleted, or misplaced, which in turn can lead to copy number variations (CNVs). Epigenetic changes affecting chromatin conformation, such as DNA methylation or histone modifications, can also arise. Paired-end sequencing offers a means to obtain relatively comprehensive descriptions of all of this somatic variation [20].

Even so, a single snapshot of a genome can only provide so much information. Modern sequencing techniques now enable analysis of spatial and temporal effects. For example, samples can be taken from different locations in a patient, either within a tissue or including primary tumor and distant metastases [18,19]. Such sampling can also include a time series, in which, for example, samples before and after treatment or during initial and relapse presentation can be used to investigate how genetics correlates with clinical protocols or outcome [17]. Although direct sequencing of samples is now routinely carried out, mutation signals from small subsets of cells are difficult to detect. Deep sequencing can mitigate these difficulties somewhat [21–23], but alternative techniques are also becoming available. For example, single-cell sequencing is now possible [24,25], although the signal obtained is relatively noisy and these experiments are currently best combined with the information gleaned from standard multicellular sequencing protocols. Alternatively, ultrasensitive methods that can detect circulating tumor DNA from plasma samples are also possible [26]. Finally, experimental techniques other than sequencing, typically not genome-wide but some single-cell–based, have also been applied (for example, fluorescence techniques [26–28]).

There have been concerted international efforts over the last few years to produce comprehensive libraries of cancer genome data across a range of tissues, including The Cancer Genome Atlas (TCGA) (http://cancergenome.nih.gov/) and the International Cancer Genome Consortium (ICGC) (https://icgc.org/). Both have collated open-access data for hundreds to thousands of samples available to the cancer research community for further study. The generation of these great volumes of data of different types is inevitably leading to a range of computational and statistical challenges.

## Data Integration and Functional Interpretation

Driver mutations are those mutations that contribute to causing cancer, as opposed to noncausal passenger mutations. Moreover, cancer genes are genes that can carry driver mutations or can contribute to oncogenesis when epigenetically modified. NGS provides increasingly better means to identify cancer genes as well as driver mutations, which has implications on the identification of biomarkers as well as on our ability to study somatic evolution of cancer on predefined cancer genes.

From sequenced tumor genomes, cancer genes and driver mutations can be predicted through the application of statistical methods for detecting overrepresentation, based on the assumption that genes that are frequently mutated across a tumor collection are likely to carry driver mutations. However, detecting recurrent mutations is challenging because the background mutation rate has been shown to be quite heterogeneous across genomes. For instance, genes with lower expression and those replicated late during the cell cycle have a higher mutation rate than genes with higher expression and those replicated early [29]. A review of methodologies for the identification of driver mutations can be found in [30].

As is frequently the case in biological analyses, when attempting to understand somatic evolution of cancer, the pathway level is perhaps more relevant than the gene level. The intuitive reason is that phenotypes providing a selective advantage to a cell are often the effect of a pathway rather than an individual gene. Consequently, a mutation in any of the genes of the pathway may provide more or less the same effect on the tumor and, hence, also a similar selective advantage. This phenomenon complicates inference and representation of how a cancer progresses towards increased malignancy. However, it also means that mutations belonging to the same pathway will tend to appear in a mutually exclusive manner, an observation that has been taken advantage of in order to identify the pathways [13,31–34], even though mutual exclusivity might also have other reasons. Predefined biological networks can, for the same reason, assist the identification of cancer genes. If pathways have a tendency to occur as subnetworks with small radius, then statistical methods can be designed for identifying such gene groups with an overrepresentation of mutated genes [35]. Several aspects of pathway and network analysis in computational cancer research are reviewed in [36].

## Intratumor Phylogeny

In principle, the genomic diversity observed within each individual tumor can reveal the evolutionary history of the tumor (Fig 1). This perspective is promising, as tumor phylogenies would allow for assessing the mode of tumor evolution and for distinguishing different hypotheses about this process. For example, monoclonal and polyclonal evolution, mutator phenotype, and cancer stem cells all leave characteristic evolutionary traces and result in distinct tumor phylogenies [37].

In practice, however, the intratumor phylogeny problem is challenging. Sampling individual cancer genomes from a tumor involves either single-cell approaches or bulk sequencing of a heterogeneous sample. Single-cell analysis seems natural for assessing genetic tumor diversity, and because of technological advances, single-cell sequencing is likely to become the state of the art soon [24,25]. However, the technology is just emerging, and large, unbiased samples are costly and still difficult to obtain. The increased levels of noise associated with amplifying individual genomes pose additional challenges on the statistical analysis of genomic data obtained in this manner [38,39]. As an alternative to exome- or genome-wide sequencing, more targeted approaches, such as fluorescent in-situ hybridization (FISH), can be used to measure specific mutational patterns in single cells [26].

On the other hand, bulk sequencing of a mixture of cells is much more robust, but it provides only indirect and imperfect evidence of the individual genomes. The deconvolution problem of grouping genomic variations into an unknown number of tumor subclones and normal cells is particularly challenging for short-read sequencing data, which complicates and often prohibits the phasing of genetic alterations. To address this problem, Bayesian approaches based on the stick-breaking process are commonly used to hierarchically cluster SNVs into clones, or genotypes, according to their estimated frequency in the tumor cell population [40,41]. For tree reconstruction, a perfect phylogeny is usually assumed, i.e., mutations are irreversible and each mutation can occur at most once in the tree. With these assumptions, the estimated SNV frequencies provide information on the tree topology. For example, if the relative frequencies of two clones sum to more than 100%, then the clone with higher frequency must be an ancestor of the one with lower frequency [42–44].

Besides SNVs, CNVs are frequent genomic changes in cancer genomes. Reconstructing tumor phylogenies from CNV data is challenging for two reasons. First, CNVs do not occur independently along the genome because they are the result of genomic alterations, such as insertions and deletions, that can affect large chromosomal segments. These spatial correlations need to be accounted for when computing evolutionary distances between CNV profiles, for example, by defining breakpoint distances or by computing the minimal number of events necessary to transform one CNV pattern into another [45–47]. Second, at each site, SNP arrays can detect the copy numbers of both parental alleles, but their phasing across sites is difficult to determine. Because the evolutionary events occur on individual haplotypes, correct evolutionary distances can only be computed based on phased CNV profiles [48]. The phasing problem has also been addressed by using external linkage information [48] and by solving it jointly with tree reconstruction using a minimum evolution criterion [47]. Finally, CNVs can confound SNV frequencies, and both data types should be analyzed jointly to obtain a more comprehensive picture of the evolutionary history of the tumor [49,50].

## Spatial Genomics and Biogeography

It is natural to extend studies of intratumor heterogeneity by asking how the heterogeneity is distributed spatially in a single tumor and how this distribution varies across tumors. Such investigations of spatial distribution may very well bear a resemblance to biogeography, a field concerned with understanding the geographic distribution of genetic variation within species as well as among closely related species [51]. As new experimental techniques emerge that allow for increasingly fine-grained assays of genetic variation across cells of tumor cross sections, and eventually the entire three-dimensional tumor, studies of spatial distribution of heterogeneity will become increasingly feasible in computational cancer research. This trend will, for instance, make it possible to analyze tumor heterogeneity by relating and contrasting the spatial distribution with the phylogenetic distribution of tumor cells.

Already today, there are a number of spatial transcriptomics techniques, while other spatial omics techniques are emerging, such as spatial genomics and proteomics [52]. Crosetto et al. [52] speculate that the next wave of sequencing devices, such as long-read, single-molecule technologies, might integrate electrophoretic systems, moving DNA and RNA molecules from a tissue section directly to a local nanopore and thereby enabling in situ single-cell sequencing. It may also turn out to be viable to locally add barcodes to DNA and RNA molecules to annotate the original location and thereafter sequence the resulting barcoded nucleic acids in one batch. In fact, coarse-grained techniques of this type are emerging for spatial transcriptomics, and more fine-grained versions, i.e., having single-cell resolution, are bound to emerge. Independently of the technical details, it is highly likely that, in the near future, spatial-omics data will provide key insights into tumor heterogeneity.

There are four types of phylogenetic methods in biogeography: diffusion models, island models, hierarchical vicariance, and reticulate models. The latter three models use discrete areas, between which species or individuals can move. Although it may be of interest to ask whether, and potentially how, tumor cells have moved between specific, predetermined regions of a tumor, perhaps identified by a pathologist, the fact that diffusion models allow for continuous movements probably make them more applicable in tumor studies. However, the entire spatial area is in this case the growing tumor, i.e., far from being constant, which is likely to further complicate the analysis; a cell can move because of addition of other cells or because of its own movement.

In a study of tumor heterogeneity, Navin et al. [25] took a first step towards a joint analysis of spatial and evolutionary aspects. They categorized a tumor as monoclonal or polyclonal, depending on whether or not all its cells have the same genomic structure. Among the polyclonal category, they found tumors in which the genomically homogeneous subpopulations were spatially segregated but also those in which the subpopulations were intermixed. It will be highly interesting to find out how common these basic categories are, how they can be refined, and what the clinical impact of these refinements may be.

## Tumor Cell Population Dynamics

In order to describe the population dynamics of evolving tumors, different modeling approaches have been used. Population genetics models, based on the Wright–Fisher process or the Moran process, can be used to model the fate of individual cells in a population [53]. More generally, branching processes have frequently been employed to account for stochastic fluctuations in the growth and composition of the population (Fig 1) [54,55]. These stochastic models or their deterministic approximations can often be solved analytically under simplifying assumptions, which allows for computing key quantities of interest, including the probability of and time to fixation of a mutant and the size and age of the tumor cell population. By contrast, models with more intricate features, such as population structure or cellular interactions, quickly become intractable. Cellular automata are a popular choice for this model class, whose analysis relies on forward simulations [56]. Thus, simple models can provide easy-to-capture insights at the risk of oversimplification, while complex models may capture more details of the evolutionary process at the cost of being difficult to analyze comprehensively.

Population genetics modeling of cancer has addressed many aspects of this somatic evolutionary process, including tumor initiation, tumor progression, and drug resistance development. Tumor initiation models aim at identifying the rate-limiting steps in the first transformations of a normal cell. Early in cancer research, a dichotomy was identified between (1) oncogenes that, when gaining increased activity through mutations, directly promote cancer (by enhancing the ability of the cell to grow and proliferate, for example) and (2) tumor suppressor genes that normally protect against cancer (by initiating programmed cell death upon signs of uncontrolled growth, for example). Tumor initiation models have highlighted the different dynamics of oncogene activation versus tumor suppressor gene inactivation, the role of chromosomal instability, and the importance of the spatial structure of the tissue of origin [57].

Tumor progression models focus on the process of mutation accumulation and further neoplastic transformation in an initiated tumor. These models have been used to infer the velocity at which mutant waves sweep through the cancer cell population and to elucidate how this speed of adaptation depends on the mutation rate, the fitness advantage of driver mutations, and on the feasible set of mutational pathways [58–60]. The tumor progression dynamics can also inform the discrimination of driver from passenger mutations [61,62], as driver mutations are commonly predicted by detecting genes under positive selective pressure. Evolutionary models can quantify the probability of any mutation to reach fixation in a tumor cell population, including advantageous drivers as well as neutral or even deleterious passengers hitchhiking on advantageous clones [63].

Mathematical models of drug resistance development date back to Luria and Delbrück [64], who studied viral resistance in bacteria, and Goldie and Coldman [65], who analyzed drug resistant tumor subclones. The key conclusion of these and other studies is that drug resistance mutations are much more likely to preexist in the tumor prior to treatment, as opposed to being generated under treatment. The probability of a preexisting single-gene mutation is generally high, such that resistance against any drug targeting a single gene can be expected to be implanted in any large tumor [66,67]. These predictions have been confirmed repeatedly. For example, in colorectal carcinomas, resistance to epidermal growth factor receptor (EGFR) inhibitors has recently been observed after a fairly constant time period, supporting the notion that resistance is a fait accompli [12]. These dynamics of evolutionary escape from selective drug pressure suggest that long-term tumor suppression can only be achieved by therapies targeting more than one pathway. Indeed, using branching processes, it has been predicted that combination therapies have a considerably higher probability of success than sequential monotherapies of the same drugs [68].

Mathematical modeling of tumor cell population dynamics will result in models and software tools that are potentially predictive of disease progression and treatment outcome. At present, much of the large-scale sequencing that has taken place is of limited depth and geographical scope, and mathematical models based on such information will likely be crude and approximate. To construct realistic models of the evolutionary processes taking place, we need to combine models that are representative of the processes taking place with high-resolution data. In the long run, ecological models of the entire tumor microenvironment may play this role [69]. The best current candidate for an approach is single-cell sequencing. By utilizing such data, in both spatial and temporal capacities, along with information obtained in parallel experiments, such as ultra-deep sequencing of circulating tumor DNA, exosomes, or cells, an accurate picture of tumor heterogeneity and evolution dynamics will potentially be realized. To achieve this with decent precision will likely require information from thousands of individual cells. Furthermore, to gain an appreciation of the variation of these processes across different tumors will require such data from patient numbers similar to the recent ICGC and TCGA consortia. Although these requirements are beyond current capabilities, continuing improvements in current technologies and the introduction of new methods such as nanopore sequencing are likely to see a climb in data resolution and will result in a need for such mathematical approaches.

## Cancer Progression Networks

**Fig 2:**
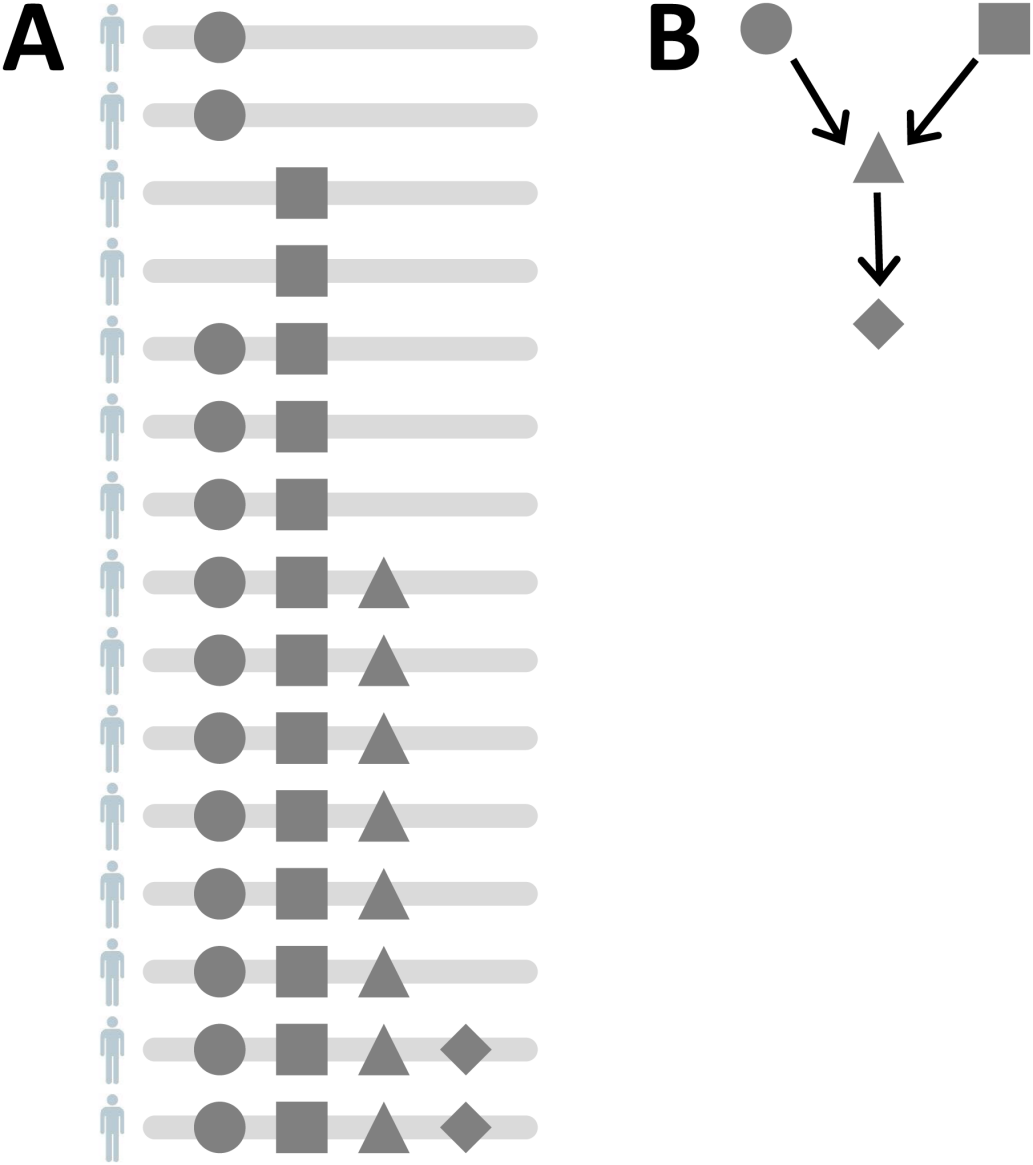
Cancer progression networks. (A) Schematic representation of cancer genomes obtained from different patients. Each row represents one patient. Four different mutations are indicated by disc (●), square (■), triangle (▲), and diamond (♦). (B) A cancer progression network that is consistent with the data shown in (A). In the directed acyclic graph, vertices are labelled by mutations, and edges indicate dependencies. Here, both mutations ● and ■ must occur before ▲ and finally ♦ can occur. Thus, the model encodes two mutational pathways, namely ● → ■ → ▲ → ♦ and ■ → ● → ▲ → ♦, and each tumor would follow exactly one of these.

Multistage theory suggests that cancer progression involves several rate-limiting events, and early on, genetic alterations have been proposed to play this role. For example, the development of colorectal cancer has been mapped to a series of genetic changes involving successive mutations in the genes *APC*, *KRAS*, and *TP53* [70]. In general, however, the diversity of genomes obtained from tumors of the same histological type is very high, complicating the identification of cancer driver genes and the discovery of biomarkers. The linear progression model initially proposed for colorectal cancer cannot explain the entire genomic diversity observed in cancer genome sequencing data today. Cancer progression models address this challenge and try to estimate common features of tumor progression across tumors of the same type. Each tumor is regarded as an independent realization of the same evolutionary process (Fig 2A).

Several cancer progression network models have been developed. They are usually formulated as probabilistic graphical models, in which a directed acyclic graph represents all feasible progression paths (Fig 2B) [71–74]. The network models generalize the linear model in several different ways. They allow for more general graph topologies, including trees, mixtures of trees, and acyclic graphs, and they account for observation errors. Most models make monotonicity assumptions, which state that, for any mutation to occur, all (or some) of its predecessor mutations in the graph need to have occurred before. This assumption makes learning the model structure from data more efficient, as compared to general graphical models. Various learning algorithms have been proposed, including exact combinatorial optimization techniques, local optimization using the structural expectation-maximization (EM) algorithm, heuristic search strategies, and Bayesian inference using Markov chain Monte Carlo (MCMC) [2,75,76].

Cancer progression models have been shown to improve prediction of patient survival. In particular, they allow for quantification of the degree of progression for each tumor, for example, as the expected waiting time for the specific mutational pattern to accumulate [77,78]. As such, progression models can be regarded as evolutionary biomarkers offering data-derived progression scores, which can complement classical tumor staging and grading.

## Summary and Outlook

Driven by technological advances in genomics, cancer research is changing rapidly. Mathematical, statistical, and computational methods play an increasingly important role in this process, and many problems that occur in today’s cancer research can be addressed with methods that are familiar to computational biologists. On the other hand, many novel modeling and computational problems arise in cancer research that lead to methodological research in computational biology. With the ongoing large-scale generation of (single-cell, spatiotemporal) genomic profiles of tumors that are publically available, computational biologists have the opportunity to make substantial contributions to cancer research. Here, we have highlighted a few major challenges which we believe are central for making progress, including (i) reconstruction of the evolutionary history of a tumor based on different types of genomic alterations, (ii) functional interpretation of mutations, and (iii) predictive modeling of the evolutionary dynamics of cancer. Statistically robust and computationally efficient methods of addressing these challenges will enable a range of applications, such as optimal control of tumor development, forecast of drug resistance evolution, and design of optimal individualized treatment strategies.
